# Ibrutinib in previously treated chronic lymphocytic leukemia patients with autoimmune cytopenias in the RESONATE study

**DOI:** 10.1038/bcj.2017.5

**Published:** 2017-02-03

**Authors:** M Montillo, S O'Brien, A Tedeschi, J C Byrd, C Dearden, D Gill, J R Brown, J C Barrientos, S P Mulligan, R R Furman, F Cymbalista, C Plascencia, S Chang, E Hsu, D F James, P Hillmen

**Affiliations:** 1 Niguarda Cancer Center, Milano, Italy; 2 MD Anderson Cancer Center, Houston, TX, USA; 3 The Ohio State University Medical Center, Columbus, OH, USA; 4 The Royal Marsden Hospital, London, UK; 5 Princess Alexandra Hospital, Brisbane, QLD, Australia; 6 Dana-Farber Cancer Institute, Boston, MA, USA; 7 Hofstra Northwell School of Medicine, Hempstead, NY, USA; 8 Royal North Shore Hospital, Sydney, NSW, Australia; 9 Weill Cornell Medical College/New York Presbyterian Hospital, New York, NY, USA; 10 Hôpital Avicenne, Paris, France; 11 Pharmacyclics LLC, an AbbVie Company, Sunnyvale, CA, USA; 12 The Leeds Teaching Hospitals, St James Institute of Oncology, Leeds, UK

Autoimmune cytopenias occur in up to 10% of patients during the course of chronic lymphocytic leukemia (CLL), with autoimmune hemolytic anemia (AIHA) the most common, followed by immune-mediated thrombocytopenia (ITP).^1, 2^ These disorders may occur at any time during disease course and depend on complex interactions between the malignant clone, impaired T-cell function, microenvironment and the immune system. Prevalence of AIHA ranges from 2.9 to 10.5%, and is generally associated with advanced disease.^3^ Clinically significant ITP occurs in ~2% of patients.^1, 2, 3^ Ibrutinib, a first-in-class, once-daily inhibitor of Bruton's tyrosine kinase (BTK), is indicated by the US FDA for the treatment of patients with CLL/small lymphocytic lymphoma (SLL), including patients with 17p deletion, and allows for treatment without chemotherapy. In addition to BTK, ibrutinib targets members of the Tec kinase family, ITK and TEC, and TXK which may impact immune function.^4^ To address the impact of ibrutinib on CLL-associated AIHA/ITP, we retrospectively analyzed data from patients in the phase 3 RESONATE study comparing ibrutinib versus ofatumumab in previously treated CLL, including patients with history of ongoing complications of AIHA and/or ITP.^5^

History of AIHA and/or ITP was collected as complications of CLL, including status at study entry (ongoing/resolved), from 386 patients who received study treatment for this analysis (*n*=195 ibrutinib; *n*=191 ofatumumab). Per the study protocol, AIHA was defined by at least one marker of hemolysis (indirect bilirubin above the upper limit of normal (ULN) not due to liver disease, increased lactate dehydrogenase (above ULN) without alternative etiology, or increased absolute reticulocytes (above ULN) or bone marrow erythropoiesis in the absence of bleeding) and at least one marker of autoimmune mechanism (positive direct antiglobulin for IgG or C3d, cold agglutinins).^6^ ITP was defined by platelets ⩽100 000 per μl and increased megakaryocytes on bone marrow exam. Patients with uncontrolled AIHA or ITP (defined as declining counts within the screening period or requirement for steroids >20 mg daily) were excluded from the RESONATE study. Other patients with AIHA/ITP including those meeting IWCLL 2008 criteria for treatment were eligible.^7^ Standard supportive care medications were permitted per protocol.

Protocol-defined AIHA and ITP were reported at the discretion of the investigator at study entry based on assessments made during the screening period. Baseline hemoglobin and platelet counts for the present analysis were based on measurements from the first day of study treatment. Hemoglobin and platelet counts over time were assessed in patients with ongoing AIHA (*n*=21 ibrutinib; *n*=9 ofatumumab) and ongoing ITP (*n*=12 ibrutinib; *n*=10 ofatumumab) at study entry, respectively. Corticosteroid use for autoimmune complications and treatment-emergent adverse events (AEs) of AIHA and ITP were collected for all treated patients. Ofatumumab data were censored at initiation of crossover to ibrutinib. Ofatumumab regimen was limited to 6 months; AE reporting period for this arm was complete at publication of interim analysis.^5^

Status of AIHA and ITP associated with CLL at study entry is shown in Table 1. At baseline, patients on ibrutinib with ongoing AIHA had median hemoglobin of 10.4 g/dl (range: 8.1–13.7), which increased to 12.4 g/dl (range: 10.3–14.6) at 24 weeks (Figure 1a). Patients on ibrutinib with ongoing ITP had median platelet count of 49 × 10^9^ per liter (range: 20–138 × 10^9^) at baseline compared with 94 × 10^9^ per liter (range: 64–248 × 10^9^) at 24 weeks (Figure 1b). In patients on ofatumumab, median hemoglobin for patients with AIHA and platelet counts for patients with ITP were 10.2 g/dl (range: 7.3–12.9) and 66 × 10^9^ per liter (range: 23–126 × 10^9^), respectively, at baseline, and 13.9 g/dl (range: 11.1–15.5) and 115 × 10^9^ per liter (range: 58–186 × 10^9^), respectively, at 24 weeks. Baseline hemoglobin in patients with AIHA was similar between arms. Baseline platelet count in patients with ITP was lower with ibrutinib than ofatumumab. Median hemoglobin level and platelet counts improved early following ibrutinib initiation and were generally sustained throughout a median 18.9 months of follow-up. Although data were limited for the patients with ongoing AIHA in the ofatumumab arm, it appears that these patients also experienced some improvement in hemoglobin levels during the follow-up.

For all treated patients (*n*=195 ibrutinib; *n*=191 ofatumumab), median treatment duration (reflecting AE follow-up) was 18.3 months for patients on ibrutinib versus 5.3 months for ofatumumab. Median treatment duration for patients on ibrutinib with ongoing AIHA and ITP at study entry was 17.7 and 17.3 months, respectively. Five ibrutinib patients with ongoing AIHA were receiving concomitant corticosteroids for autoimmune cytopenias (AIC) at baseline; one discontinued corticosteroids on day 42 of therapy. One ofatumumab patient with ITP was receiving concomitant corticosteroids for autoimmune complications at baseline and during the treatment period. Corticosteroid use was initiated for AIC in four patients on ofatumumab, compared with one on ibrutinib who had prior medical history of AIHA.

The AE profile for patients with ongoing AIHA/ITP randomized to ibrutinib (Supplementary Table 1) was similar to the overall ibrutinib population. Among all treated patients, two developed AIHA and two developed ITP; all four were randomized to the ofatumumab arm. Of the two patients with AIHA, one was treated with prednisolone for 16 days and was ongoing at time of analysis; the other was hospitalized and treated with transfusion, prednisolone, folic acid, and eventually splenectomy. Of the two patients with ITP, one received platelets, and the ITP resolved; the other had a longer course of ITP that required intravenous immunoglobulin, steroids and platelets. No new AICs were reported as an AE in patients randomized to ibrutinib.

Treatment-emergent AIC has long been recognized as a possible complication of CLL. Initially, most of the data reporting on AIC in patients receiving ibrutinib have been described in case reports. Several cases showed activity of ibrutinib in controlling steroid-refractory AIHA in high-risk del17p CLL,^8, 9^ or cessation of sequential episodes of severe AIHA/ITP and Coombs test negativity in a patient with similar prognostic features.^10^ Interestingly, acute recurrence or ‘flare' of AIC following ibrutinib initiation has been reported in a series of CLL patients.^11^ The majority of these patients continued to receive ibrutinib with or without the addition of other therapies (most commonly steroids and IVIG) to treat the autoimmune complication, which led to resolution or control of AIC in most patients in this series.^11^ To explore the effect of ibrutinib on AIC, Rogers *et al.*^12^ retrospectively collected data on 301 patients enrolled in four sequential clinical trials. Of 22 patients receiving therapy for AIC during ibrutinib treatment, 19 were able to discontinue immunosuppressive therapy (including but not limited to corticosteroids). Furthermore, ibrutinib was associated with a low rate of treatment-emergent AIC.^12^

Data from our study in a cohort of patients at increased risk for AIC corroborate findings that ibrutinib does not precipitate recurrence of AIHA/ITP and can be administered in patients with previous history of these complications. In our analysis, ofatumumab did not lead to exacerbation or decrease in blood counts in the majority of treated patients, likely due to the fact that CD20 monoclonal antibodies themselves have long been considered as adjunct therapy for AIC. These observations are notable in contrast with the history of purine analog therapies such as fludarabine, which have been known to trigger the development of AIHA/ITP, particularly in monotherapy. Therapy-triggered AIHA has also been reported in 1–5% of CLL patients treated with fludarabine-containing combination regimens.^1^ In our study, no new cases of AIHA or ITP occurred in patients on ibrutinib with a median treatment duration of 18.3 months, compared with four patients on ofatumumab with a median treatment duration of 5.3 months.

Limitations of these analyses include that history of autoimmune process was reported without serially monitoring serologic findings associated with these events, and uncontrolled AIHA or ITP was not assessed, though this population is frequently excluded in studies of CLL.^12^ Further, this study did not contain patients who solely were experiencing AIC as their only manifestation of CLL, as all patients had measurable lymphadenopathy and the majority had other manifestations of active or progressive CLL. Although mechanisms of autoimmune disease in CLL are not extensively understood, a potential role for FcγR signaling has been noted in autoimmune arthritis models implicating BTK inhibition in the suppression of BCR and FcγR signaling.^13, 14^ Additional evidence suggests that AIC may be mediated by polyclonal B- and T-cell responses rather than by the malignant B-cell clone.^15^ Further elucidation of a putative FcR-based mechanism and other underlying mechanisms in inflammatory autoimmune disorders such as AIHA/ITP will aid in CLL treatment decision-making and contribute to our understanding of the beneficial effects of ibrutinib.^13^

## Figures and Tables

**Figure 1:**
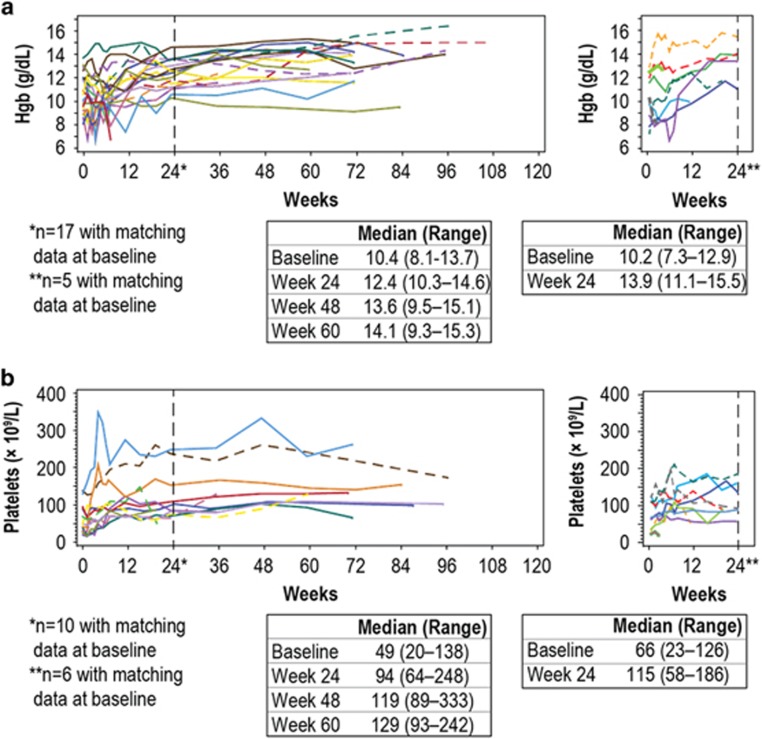
Hemoglobin and platelet counts in patients on ibrutinib or ofatumumab with ongoing AIHA and ITP. (**a**) Hemoglobin levels in patients on ibrutinib (left) or ofatumumab (right) with ongoing AIHA at study entry. (**b**) Platelet counts in patients on ibrutinib (left) or ofatumumab (right) with ongoing ITP at study entry. Dotted vertical line indicates the 24-week time point. Complete blood count with differential was collected weekly for the first 8 weeks, every 4 weeks until week 24, every 12 weeks until week 72, and every 24 weeks thereafter. Weeks 84 and 108 were not scheduled visits.

**Table 1:** Status of AIHA and ITP complications in patients receiving ibrutinib or ofatumumab

*Autoimmune cytopenia*	*Ibrutinib (*n*=195)*	*Ofatumumab (*n*=191)*
History of either AIHA or ITP, *n* (%)	38 (19.5%)	42 (22%)
*AIHA*		
Ongoing at study entry, *n* (%)	21 (10.8%)	9 (4.7%)
Resolved prior to study entry, *n* (%)	5 (2.6%)	18 (9.4%)
Adverse event on therapy, *n* (%)	0	2 (1%)^a^
		
*ITP*		
Ongoing at study entry, *n* (%)	12 (6.2%)	10 (5.2%)
Resolved prior to study entry, *n* (%)	4 (2.1%)	8 (4.2%)
Adverse event on therapy, *n* (%)	0	2 (1%)^b^
		
*Both AIHA and ITP*		
Ongoing at study entry, *n* (%)	8 (4.1%)	6 (3.1%)

Abbreviations: AIHA, autoimmune hemolytic anemia; ITP, immune-mediated thrombocytopenia.

a AIHA reported as a grade 3/4 event in one patient.

b ITP reported as a grade 3/4 event in both patients.
