# Supplementary material for: Ibrutinib in previously treated chronic lymphocytic leukemia patients with autoimmune cytopenias in the RESONATE study
Source: Blood Cancer J. 2017 Feb 3;7(2):e524–. doi: 10.1038/bcj.2017.5 (PMC5386339; doi:10.1038/bcj.2017.5)
Supplement: Supplementary Table 1 [file bcj20175x1.docx]

**Supplementary Information**

**Supplementary Table 1. Most frequent treatment-emergent adverse events in patients receiving ibrutinib with ongoing AIHA or ITP**

| **Adverse Event** | **AIHA**  **(n=21)** | **ITP**  **(n=12)** |  |
| --- | --- | --- | --- |
|  | Number of patients | | |
| Any grade |  |  | |
| Diarrhea^*^ | 11 | 9 | |
| Pyrexia^†^ | 10 | 6 | |
| Grade 3/4 |  |  | |
| Neutropenia | 4 | 3 | |
| AIHA, autoimmune hemolytic anemia; ITP, immune-mediated thrombocytopenia.  ^*^Grade 3/4 in 1 patient with ITP.  ^†^Grade 3/4 in 1 patient with AIHA. | | | |
